# Post-Translational Modifications and Functional Heterogeneity of Human Serum Albumin Formulations

**DOI:** 10.3390/toxins18100418

**Published:** 2026-09-28

**Authors:** Roko Gladović, João G. Brás, Jeroen C. Vollenbroek, Karin G. F. Gerritsen

**Affiliations:** Artificial Kidney Innovation Lab., University Medical Center Utrecht, 3584 CX Utrecht, The Netherlands; roko.gladovic@gmail.com (R.G.); j.c.vollenbroek@umcutrecht.nl (J.C.V.)

**Keywords:** human serum albumin, post-translational modifications, albumin dialysis

## Abstract

Human serum albumin (HSA) is the most abundant plasma protein and plays essential roles in regulating oncotic pressure, transporting endogenous and exogenous compounds, and maintaining redox balance. Because of its biochemical properties, HSA is widely used in clinical medicine, biotechnology, and pharmaceutical development. However, albumin is not a static molecule. It undergoes numerous post-translational modifications (PTMs), including oxidation, glycation, carbonylation, carbamylation, and aggregation, which can significantly alter its structure, ligand-binding properties, antioxidant capacity, and pharmacokinetics. Furthermore, commercial albumin products exhibit substantial heterogeneity arising from differences in source material, manufacturing processes, formulation conditions, and storage practices. This review summarizes the current understanding of PTMs affecting human serum albumin, with particular emphasis on their molecular origins and functional implications. We discuss how physiological, pathological, and manufacturing-induced modifications influence albumin activity and contribute to variability among commercial albumin products. In addition, we examine established and emerging applications of PTM-modified albumin in diagnostics, critical care, extracorporeal detoxification, drug delivery, nanomedicine, and regenerative medicine. Finally, we highlight current challenges related to product heterogeneity, quality control, regulatory assessment, and discuss future opportunities in albumin-based technologies. A deeper understanding of albumin PTMs is essential for improving critical care and developing next-generation albumin-based biomedical products.

## 1. Introduction

Human serum albumin (HSA) is the most abundant human plasma protein, renowned for its multifaceted physiological roles, including maintenance of oncotic pressure, transport of endogenous and exogenous molecules, antioxidant activity, and modulation of inflammation [1,2,3]. Over the past decades, the clinical use of HSA has expanded from simple volume replacement to more sophisticated applications such as drug delivery, detoxification therapies, diagnostics, and tissue engineering [4,5,6,7]. Nowadays, because of its exceptional stability (half-life of 19 days [8]), solubility, and binding capacity, albumin is widely used in clinical medicine (as a plasma volume expander and detoxifying agent) [9,10], and in biotechnology (to support cell culture, stabilize biologics, and enable drug-delivery strategies) [4,11]. Central to these advances is the recognition that HSA is not a static molecule: it undergoes a wide array of post-translational modifications (PTMs) in both healthy and diseased tissues, as well as during manufacturing processes [12,13,14,15]. These PTMs—such as oxidation, glycation, carbamylation, carbonylation, succinylation, and more—can profoundly alter HSA’s structure and function [14,15,16,17,18].

Commercially available albumin mixtures display heterogeneity. Differences in source material (plasma-derived or recombinant), fractionation and purification methods, pasteurization/virus inactivation steps, buffer composition and pH, mixture concentration (5%, 20%, or 25%), and the addition of stabilizers (sodium caprylate and sodium *n*-acetyl-tryptophanate (*n*-AcTrp)) create distinct molecular fingerprints that alter the functional properties of albumin [19,20,21]. More specifically, differences in manufacturing and formulation processes lead to differences in the amount of oxidized HSA [18], polymer/dimer content [22], fatty acid (FA) occupancy [23], and other attributes, which may affect the functionality of the mixture and potentially have clinical implications.

Despite multiple analytical and product-specific studies [18,19,22], systematic, standardized comparative data across marketed albumin products remain limited. Consequently, there is no unified framework for predicting how a particular modification will affect function in each application. This review synthesizes current knowledge on PTM-modified and functional heterogeneous human serum albumin formulations in medicine—how these modifications occur, and which are most prevalent. Finally, this review provides perspective on how post-translational modifications shape the molecular and functional properties of human serum albumin and their potential relevance to its therapeutic applications. This narrative review was informed by a literature search in PubMed, Elsevier, and Google Scholar. Search terms included combinations of “human serum albumin”, “commercial human serum albumin”, “albumin formulation”, “albumin preparation”, “post-translational modification”, “oxidation”, “glycation”, “aggregation”, “redox state”, “ligand binding”, and related terms relevant to the topics covered in this review. Reference lists of relevant original articles and review papers were additionally screened to identify further pertinent publications. Articles were selected based on their relevance to the scope of the review, with particular emphasis on studies addressing post-translational modifications, manufacturing- and formulation-related differences, functional properties, and clinical or biotechnological applications of HSA.

## 2. Molecular Basis: Structure and Functional Impact of Human Serum Albumin Post-Translational Modifications

### 2.1. Structural and Functional Overview of Human Serum Albumin

HSA is a single-chain, non-glycosylated protein of 585 amino acids with a molecular weight of ~66.5 kDa [7,24]. Its structure comprises three homologous domains (I–III), each subdivided into A and B subdomains, as shown in Figure 1. The three domains create a heart-like overall fold that characterizes albumin’s stability and ligand-binding versatility [25]. The prominent binding sites are named Sudlow site I and Sudlow site II [25]. They are located in subdomains IIA and IIIA, respectively, and bind a plethora of drugs, fatty acids, metabolic waste products, and other small molecules [25].

Human serum albumin plays a part in many mechanisms, such as the regulation of oncotic pressure, as a carrier for many organic ligands, and the regulation of the oxidation state [26]. The primary role of albumin in circulation is to provide colloidal osmotic pressure (COP). As it is quite abundant in plasma (~40 g/L) and has a medium molecular weight, this protein contributes to approximately 80% of total COP [26]. HSA synthesis in the liver is responsive to COP through feedback regulation of hepatic albumin gene expression, thereby maintaining homeostasis [27]. Secondly, HSA carries a broad spectrum of endogenous and exogenous compounds [28], including FAs, bilirubin, hormones, metal ions, uremic toxins (the so-called ‘protein-bound uremic toxins’ (PBUTs)) [29], and medicinal drugs [25,28]. Its strong ligand-binding capacity is critical for the distribution, availability, and clearance of the named compounds. Finally, its third prominent role is linked to its antioxidant capacity—albumin’s Cys-34 free thiol can scavenge reactive oxygen species (ROS) or undergo redox reactions, helping maintain redox balance in plasma [30].

### 2.2. Functional Consequences of Human Serum Albumin Redox States and Other Types of Post-Translational Modifications

The presence of numerous lysine residues and a single free cysteine (Cys-34) makes HSA particularly susceptible to diverse PTMs [14,16]. Most importantly, Cys-34 is the only free cysteine residue and the only free thiol in HSA under physiological conditions. While all cysteine residues affect the protein state, Cys-34 plays a crucial role in determining the protein redox state [30]. To start, redox alterations at Cys-34 shift HSA between three isoforms (Figure 2) [30]: human mercaptalbumin (HMA), the free thiol form of HSA, constituting around 70% of total HSA in the body [30]; human non-mercaptalbumin-1 (HNA-1), a mixed disulfide with a small thiol compound, such as Cys, cysteinglycine (CysGly), homocysteine (HCys), or GSH, constituting approximately 25% [30]; and non-mercaptalbumin-2 (HNA-2), the higher oxidized form of the cysteine residue as sulfinic or sulfonic acid, accounting for approximately 5% of total HSA [30].

The redox state of Cys-34 is a critical determinant of the protein’s antioxidant function [18], and increased levels of HNA forms correlate negatively with these antioxidant properties [31]. Oxidation causes subtle conformational changes that can also alter albumin’s ligand-binding affinity to certain compounds [31], such as protein-bound uremic toxins (PBUTs). Specifically, binding affinities for certain endogenous ligands (bilirubin and tryptophan) and exogenous drugs (warfarin and diazepam) decrease in proportion to HSA oxidation (cysteinylation on Cys-34). Furthermore, binding affinities for lipid mediators also differ between reduced and oxidized HSA isoforms. Proatherosclerotic lipids have higher affinities for oxidized HSA, while anti-atherosclerotic mediators possess higher affinities for reduced HSA isoforms [30]. Increased levels of endogenous HNA are linked to liver cirrhosis, chronic kidney disease (CKD), and diabetes mellitus [30]. Observational studies have reported associations between albumin redox status and cardiovascular outcomes in dialysis patients [32]. In hemodialysis patients, an HNA fraction > 51.16% was associated with a 2.22-fold higher adjusted risk of cardiovascular mortality [33], while a small study in peritoneal dialysis patients found lower HMA levels in patients who experienced cardiovascular events [34].

Recent proteomic studies have cataloged over 120 novel PTMs on albumin, including O-phosphorylation, glycation (non-enzymatic addition of sugars), methylation, carbonylation (oxidative modification), acetylation, succinylation, crotonylation, beta-hydroxybutyrylation, lactylation, nitrosylation, palmitoylation, geranylation, farnesylation, and more [15,16]. Many modifications occur at or near ligand-binding regions or Cys-34 [14,15], directly impacting albumin’s transport capacity or redox properties. For example, palmitoylation may affect fatty acid binding sites [23]. Farnesylation and geranylation may affect albumin interaction with small molecules and drugs, as these PTMs may interfere with binding in the Sudlow II region [23]. The location of myristoylation at the C-terminus of albumin may interfere with the control of albumin stability [23]. Glycosylation can prolong half-life but may affect tissue distribution or clearance [35]. Carbonylation is associated with permanent loss of HSA function and increases VCAM-1 and ICAM-1 expression, which may promote atherogenesis [18,36]. O-glycosylation by Glc-NAc, phosphorylation, octanoylation, and dehydroalanine PTMs were observed at Sudlow sites and one of the fatty acid binding sites [23]. PTMs frequently coexist and may influence one another’s formation and functional consequences. For instance, glycation can generate AGEs and promote secondary oxidative reactions, while glycation-induced conformational changes can alter the accessibility and reactivity of Cys-34 [37]. Oxidation may promote aggregation. Aggregation can subsequently reduce ligand-site accessibility and alter protein conformation. Importantly, the relevance of these PTMs and their interaction to commercial HSA formulations remains to be established.

In commercial albumin mixtures, protein aggregation is an ever-present issue that must be monitored, as high levels of aggregates could pose an immunogenic risk and impair the ligand-binding and transport functions of albumin. Commonly, commercial mixtures contain between 2% and 3% of albumin dimers [22]. Pasteurization (60 °C for 10 h), a mandatory step for viral inactivation, is the primary stressor causing aggregation [38]. Heat-induced partial unfolding exposes hydrophobic regions, promoting intermolecular interactions and disulfide cross-linking, resulting in polymerization. Aggregation increases in pathological conditions, including liver cirrhosis and chronic liver disease (CLD) [39]. It also reduces the accessibility of ligands to binding sites [40], hindering pharmacokinetics.

Another major modification that occurs on HSA is non-enzymatic glycation [41]. In conditions of elevated glucose (e.g., diabetes) [41] or during in vitro storage, HSA can undergo non-enzymatic glycation (the Maillard reaction) [41,42]. Glycation modifies amino residues and can alter charge, conformation, and binding site topology, as well as affect ligand and drug binding [41,42]. Glycation is a non-enzymatic reaction in which reducing sugars covalently bind to lysine residues on albumin, a process accelerated by heat and prolonged storage. Long-term storage of commercial products can lead to significant accumulation of advanced glycation end-products (AGEs) [31]. Plasma-derived albumin naturally contains some glycated forms. Recombinant HSA (rHSA) products often have lower glycation levels, but modifications accumulate during processing and storage [43]. Glycation increases charge heterogeneity and can modify or block key ligand-binding sites (Sudlow sites I and II). It also prolongs protein half-life [42]. These changes may alter the pharmacokinetics and pharmacodynamics of co-administered drugs [31]. Moreover, an increase in albumin glycation is associated with diabetes mellitus, neuropathies, and coronary artery disease (CAD) [42].

Several albumin PTMs associated with pathological conditions, particularly chronic kidney disease (CKD), are likely of limited relevance to commercial albumin products, although trace levels may theoretically be present in plasma-derived HSA. For example, carbamylation, a non-enzymatic reaction of isocyanic acid (in equilibrium with urea) with protein amino groups, occurs under urea-rich conditions and has been shown to alter albumin structure, promoting amyloidogenic aggregation [17]. Similarly, guanidylation is increased in CKD and significantly reduces albumin binding capacity for ligands such as indoxyl sulfate and tryptophan [44]. Although these PTMs provide important insights into albumin dysfunction in vivo, their occurrence in commercial albumin solutions is expected to be limited, as plasma-derived HSA is obtained from healthy donors and such conditions are not typically encountered during manufacturing or storage.

Finally, commercial albumin products differ in several important aspects. Modifications such as changes in redox state, polymerization, glycation, carbonylation, and others play a key role in albumin’s functional profile (Table 1) and may affect the safety of the HSA products. Further thorough investigation is therefore warranted, particularly regarding their impact on product quality and the potential side effects associated with their administration.

## 3. Commercial Human Serum Albumin: Production and Formulation Differences

### 3.1. Plasma Human Serum Albumin (pHSA)

Commercial HSA for therapeutic use is most often derived from pooled human plasma via Cohn fractionation [47]. In this process, plasma is subjected to a sequence of cold-ethanol precipitations under controlled temperature, pH, ionic strength, and ethanol concentration; albumin remains soluble when many other plasma proteins precipitate because it possesses the highest solubility and the lowest isoelectric point (pI) among major plasma proteins [48]. This method yields a crude albumin fraction separated from the bulk of immunoglobulins, clotting factors, and other plasma proteins. Over time, refinements—including chromatographic purification or additional filtration—have been introduced to improve purity and yield [48]. After fractionation, the albumin solution undergoes virus inactivation and sterilization to ensure safety for transfusion or therapeutic use, commonly via pasteurization: heating at 60 °C for 10 h in buffered solutions, often with stabilizers (caprylate) to prevent thermal denaturation [48,49]. This step significantly reduces viral risk but exposes the protein to thermal and oxidative stress, which can alter its chemical and conformational integrity [49]. Because donor plasma comes from many individuals, pooled plasma introduces biological variability—differences in glycation, redox state, fatty-acid load, native ligand occupancy, and other PTMs. Combined with variations in fractionation parameters, solvent conditions, storage, and pasteurization protocol, this results in substantial batch-to-batch heterogeneity in commercial albumin, even from a single manufacturer.

This heterogeneity has been most thoroughly studied for oxidative modifications to human serum albumin. Oxidation of Cys-34 is influenced by the manufacturing process, particularly heat treatment (pasteurization) in the presence of dissolved oxygen. Although stabilizers can partially mitigate this effect during processing and storage [18], increased Cys-34 oxidation is frequently observed in commercial HSA preparations. For example, commercial HSA of Japanese origin showed significantly elevated HNA-1 and HNA-2 levels compared to both healthy and diseased individuals [18]. Similarly, Bar-Or et al. reported markedly higher Cys-34 oxidation in six commercial HSA products from the US and Europe compared to healthy individuals (57.2 ± 3.3% vs. 22.9 ± 4.8%) [50]. Comparative analyses further highlight manufacturer-dependent variability. Nakae et al. reported significant differences in HMA and HNA-2 content between HSA preparations from two manufacturers (HMA: 48.03% vs. 38.18%, and HNA-2: 13.20% vs. 25.07%) [51]. Together, these findings indicate substantial manufacturer-dependent variability and a mismatch in redox composition between endogenous and therapeutic albumin preparations, although the clinical relevance remains unclear. Standardization of the HMA/HNA ratio may be used as a relevant quality indicator.

Purification and formulation protocols differ among manufacturers (Table 2) [20], contributing to differences between commercial albumin products. Although Cohn fractionation remains the predominant method for plasma-derived HSA production, alternative purification strategies, such as ion exchange chromatography (IEC) and other chromatographic approaches, have been developed to improve purity and process efficiency. Alternative biological sources, such as hemolyzed human placenta blood, have also been explored because of their high HSA content (Table 2). More advanced purification techniques, such as simulated moving bed chromatography (SMB), may offer further opportunities but are currently limited by factors such as cost, complexity, scalability, or insufficient purity for clinical or industrial applications. Further optimization of these approaches may enable more efficient and cost-effective HSA production, which is particularly relevant given the substantial variation in albumin prices between providers [52].

### 3.2. Recombinant Human Serum Albumin (rHSA)

To address the limitations of plasma-derived albumin (Table 3), recombinant HSA (rHSA) has been developed and increasingly used, especially in research and therapeutic contexts where a defined, consistent material is desired [53]. rHSA is expressed in controlled host systems (yeast, mammalian cells, and plants), purified, and formulated [53]. Because it is produced from a clonal, non-plasma source, rHSA offers major advantages. To start with, rHSA eliminates donor-related variability and lacks the donor-derived PTMs common in plasma-derived HSA [53]. Secondly, rHSA can be prepared in a delipidated, ligand-free form, which is useful when binding capacity needs to be controlled [53]. Finally, there are no plasma-derived contaminants or donor-specific pathogens if handled properly [53]. However, rHSA is not automatically ‘native-like’. The expression host, culture conditions, downstream purification, and formulation determine its final chemical state [53]. PTMs such as oxidation during manufacture, aggregation, or misfolding may still occur, particularly if purification or storage is suboptimal. Consequently, quality and consistency depend heavily on the production process and quality control [53]. In many biopharmaceutical and cell culture applications, rHSA is preferred for its more predictable and controllable profile. However, cost considerations have limited its widespread replacement of plasma-derived HSA (Table 3) [53,54].

### 3.3. Formulation Variables

Beyond source and manufacturing method, certain formulation variables also influence albumin’s characteristics. Firstly, albumin products may be formulated in different buffers (e.g., normal saline, phosphate buffer, or acetate buffer), with varying pH levels and ionic strengths. In vitro research shows that differences in buffers influence protein–protein interactions and protein stability [55]. Moreover, to protect albumin during pasteurization, storage, and transport, many manufacturers include stabilizers. Stabilizers bind to albumin and shield reactive residues (e.g., Cys-34), reduce aggregation, or stabilize the tertiary structure under stress. Typical stabilizers are fatty acid derivatives [56] or aromatic amino acid analogs [57], most commonly sodium caprylate and *n*-acetyl-tryptophanate (*n*-AcTrp) [56,57]. Caprylate binds to specific FA sites, primarily to Sudlow site II [58], inducing a stabilizing conformational shift that increases the protein’s melting temperature and prevents aggregation during heat treatment [56]. *n*-AcTrp provides additional stabilization. However, the tryptophan residues in the stabilizer are susceptible to oxidation or photodegradation, potentially forming colored by-products [57]. Residual stabilizers can interfere with sensitive research applications and may need to be removed using charcoal treatment or dialysis for specific uses. Faroongsarng et al. and Anraku et al. showed that, in vitro, stabilizer binding also alters the ligand-binding capacity, possibly competing with endogenous or exogenous ligands, and may affect redox behavior [56,59]. Furthermore, Raillon et al. reported an increase in the free fraction of indoxyl sulfate from 12% to 54% following the addition of 3 mM caprylate to HSA 500 μM [58].

In summary, due to variations in source material, manufacturing process, stabilizer use, buffer composition, and other variables, even different batches of HSA product from the same production line may differ substantially in function. These differences can hinder clinical and academic research. That is why it is important to devise universal assays for product testing (e.g., HMA/HNA ratio to check for oxidation).

### 3.4. Clinical Implications of Albumin Quality

The potential impact of albumin quality on the efficacy and safety of therapeutic applications has received little attention. During therapeutic plasma exchange, patient plasma is removed and replaced with commercial albumin solutions. In addition, albumin infusions are routinely administered in critical care and hepatology for plasma volume expansion and circulatory support. This exposes patients to exogenous albumin preparations that may contain substantial proportions of post-translationally modified or oxidized albumin generated during manufacturing and storage. Preliminary clinical observations in neurological patients undergoing repeated plasma exchange demonstrated progressive accumulation of irreversibly oxidized albumin despite preserved hepatic and renal function [60]. Notably, elevated levels remained detectable after completion of treatment, suggesting limited short-term clearance of these modified species [60]. However, the clinical relevance of these observations remains unclear and warrants further investigation. Furthermore, administration of albumin products with alterations in HSA redox state, PTMs such as guanidylation, or occupancy of ligand-binding sites by stabilizers such as caprylate may reduce binding affinity and thereby increase the free plasma concentrations of protein-bound uremic toxins, drugs, or drug metabolites. Such effects may persist until the modified albumin is cleared or the stabilizer dissociates or is cleared, respectively. A higher free fraction could potentially enhance the toxicity of protein-bound uremic toxins and alter the pharmacokinetics of protein-bound drugs, although the clinical significance of this phenomenon remains to be established. Similar considerations apply to albumin dialysis and artificial liver support systems, such as the Molecular Adsorbent Recirculating System (MARS^®^), where patients experience profound toxin accumulation. In these settings, the therapeutic efficacy of albumin may depend on its redox state and preserved ligand-binding capacity [5,12,13]. Oxidative modifications or occupancy of binding sites by stabilizers or endogenous ligands may reduce detoxification efficiency, although this has received little attention. In conventional hemodialysis, removal of strongly albumin-bound uremic toxins such as indoxyl sulfate and *p*-cresyl sulfate is severely limited by their low free fraction. Reduced albumin binding, for example through displacement by compounds such as caprylate, could therefore increase the fraction available for diffusion across the dialysis membrane and potentially enhance removal [58,61]. However, whether differences in albumin-binding properties translate into clinically meaningful changes in PBUT clearance remains unknown.

Together, these observations highlight the need for systematic evaluation of albumin quality in therapeutic applications. As functional properties, such as antioxidant capacity, ligand binding, detoxification efficiency, and immunomodulatory functions, are increasingly recognized as clinically relevant [3,13], characterization of albumin redox state, post-translational modifications, and stabilizer content may become an important component of albumin product quality assessment.

### 3.5. Post-Translationally Modified Human Serum Albumin in Therapeutic and Biotechnological Applications

The influence of PTMs extends further into pharmaceutical drug delivery, biotechnology, and regenerative medicine. Owing to its excellent biocompatibility, long circulatory half-life, and multiple ligand-binding sites, albumin has become an attractive carrier for therapeutic agents. Albumin-based nanoparticles can enhance tumor accumulation through enhanced permeability and retention effects and through receptor-mediated uptake pathways involving proteins such as gp60 and SPARC [6,24,62]. The clinical potential of albumin-mediated drug delivery is exemplified by albumin-bound paclitaxel (Abraxane^®^). Beyond exploiting its native carrier properties, albumin can be deliberately modified to tailor its pharmacokinetic and targeting properties. Modification of lysine residues provides high loading capacity but may generate heterogeneous conjugates, as HSA contains numerous reactive amino groups. Site-selective approaches targeting defined residues, including Lys-225 and Lys-414, have therefore been developed to improve conjugate uniformity and enable controlled attachment of drugs, targeting ligands, fluorophores, radionuclides, and imaging probes [63]. Similarly, glycosylation, PEGylation, and other site-specific conjugation strategies are being explored to prolong circulation, enhance tumor targeting, and optimize drug release profiles [35,64,65].

Modified and recombinant albumin also have emerging applications beyond drug delivery. High-purity recombinant albumin is increasingly used in cell culture, tissue engineering, and cell therapy, where PTM-modified albumin may influence cellular responses, biocompatibility, and product performance [4]. Albumin-based nanovesicles are being developed for gene delivery, molecular imaging, multimodal cancer therapy, and treatment of inflammatory and neurodegenerative disorders [62,66].

These applications highlight the potential value of deliberately controlling albumin modification, but also the importance of distinguishing engineered modifications from unintended changes introduced during production and storage. Manufacturing-induced PTMs, aggregation, and batch-to-batch variability can alter albumin functionality and may affect its immunogenicity [1,7,12,35,62]. Consequently, advanced analytical techniques such as top-down mass spectrometry and redox proteomics are increasingly being employed to characterize PTM profiles and establish meaningful structure–function relationships [14,67]. Improved understanding and control of these modifications may both improve manufacturing consistency and quality control and facilitate the rational development of albumin products with properties tailored to specific therapeutic, diagnostic, and biotechnological applications [68].

## 4. Conclusions

Human serum albumin remains one of the most widely used and biologically important proteins in medicine and biotechnology. Traditionally regarded as a relatively simple plasma volume expander and transport protein, HSA is now increasingly recognized as a structurally dynamic molecule whose biological activity is profoundly influenced by redox status, PTMs, aggregation, ligand occupancy, and stabilizer content. This growing understanding challenges the long-standing assumption that all albumin products are functionally equivalent and interchangeable. Indeed, commercially available albumin preparations exhibit considerable molecular and functional heterogeneity despite being marketed under the same generic designation. Differences in HSA redox state, PTMs, and ligand or stabilizer occupancy may have clinical implications through reduced antioxidant capacity or altered systemic exposure to PBUTs and drugs following albumin administration, or via reduced efficiency of albumin-based extracorporeal detoxification, although the clinical relevance remains to be determined. At the same time, advances in nanomedicine, drug delivery, regenerative medicine, and extracorporeal detoxification demonstrate that carefully controlled modifications may be deliberately exploited to tailor albumin for specific applications.

Future progress will depend on establishing robust structure–function relationships through advanced analytical techniques, including high-resolution mass spectrometry and redox proteomics. Equally important is the development of standardized functional assays capable of assessing clinically relevant properties such as antioxidant activity, ligand-binding capacity, and toxin-scavenging performance. Regulatory frameworks may benefit from incorporating molecular and functional quality attributes alongside traditional purity metrics to ensure consistent product quality and minimize potentially unfavorable functional properties.

Ultimately, human serum albumin may be better considered not as a uniform biological product but rather as a family of formulations that can differ in their molecular composition and functional properties. A deeper understanding of these properties may not only improve manufacturing and quality control of existing albumin formulations and clarify their relevance for clinical use, but may also support the development of next-generation albumin products for therapeutic, diagnostic, and biotechnological applications.

## Figures and Tables

**Figure 1:**
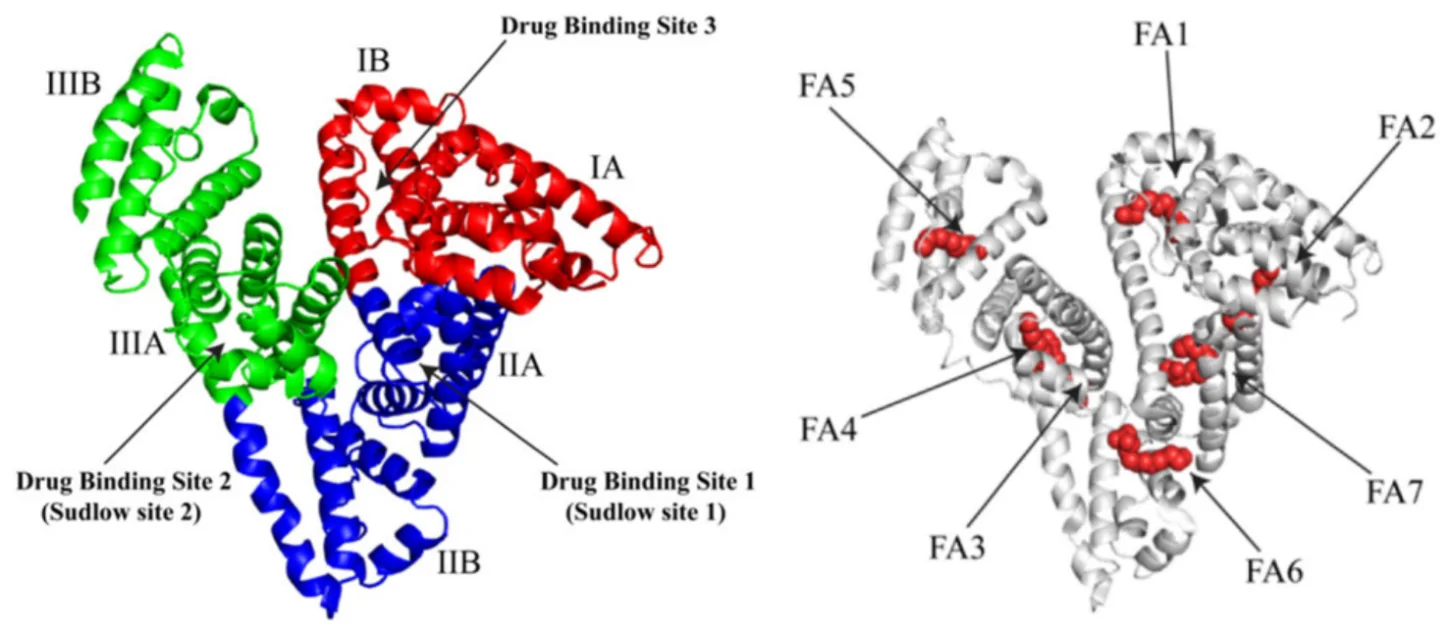
Human serum albumin structure and binding sites. Domains (domain I [in red], domain II [in blue], and domain III [in green]), subdomains (IA, IB, IIA, IIB, IIIA, IIIB), and Sudlow sites are marked (**left**). Fatty acid binding sites are marked (FA1–FA7) (**right**). Reproduced from *Mishra* et al. [25] under the Creative Commons Attribution (CC BY) license.

**Figure 2:**
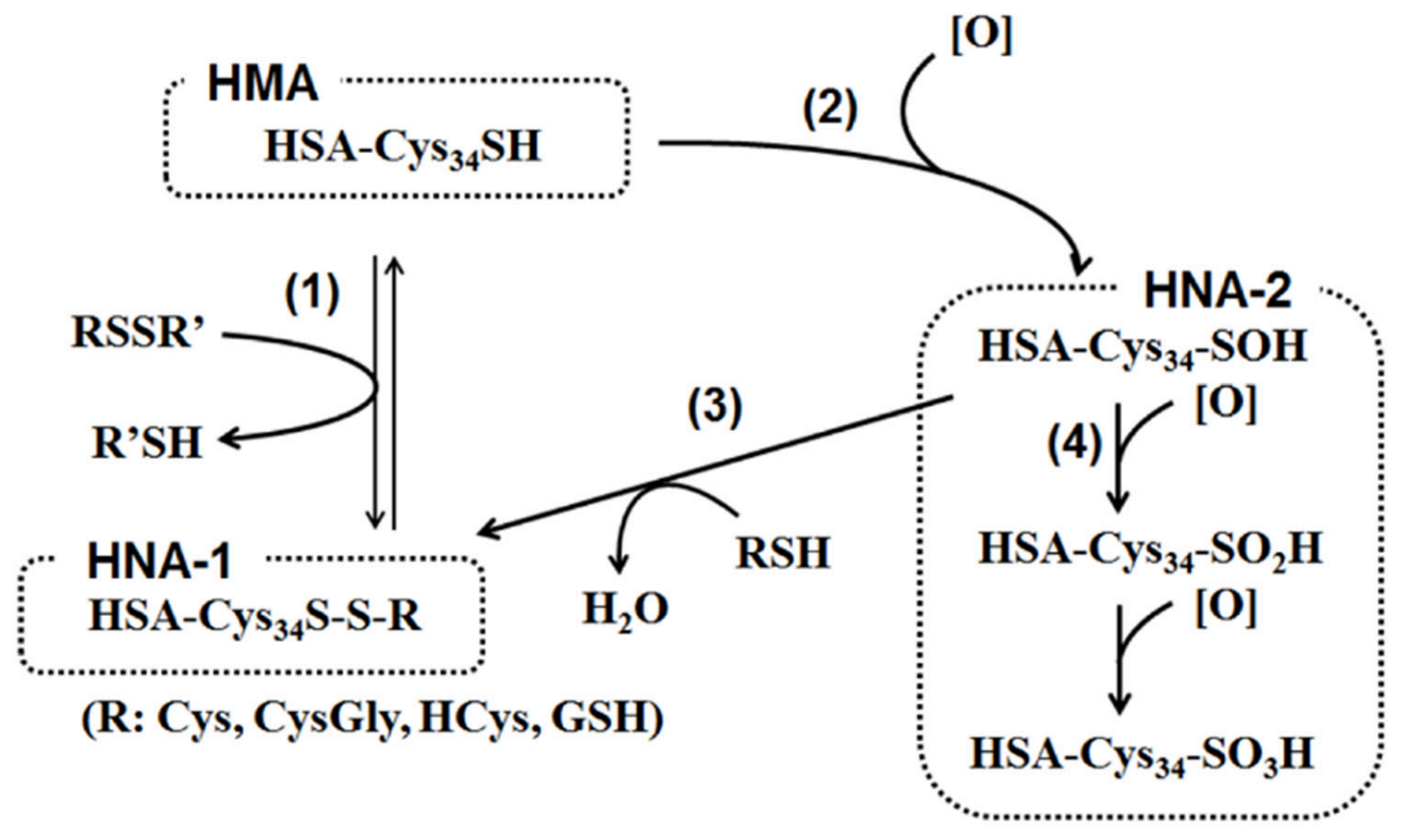
Pathway for the human serum albumin (HSA) redox state. (1) The free thiol group of Cys-34 in human mercaptalbumin (HMA) undergoes thiol–disulfide exchange with low-molecular-weight thiols (e.g., cysteine (Cys), cysteinglycine (CysGly), homocysteine (HCys), glutathione (GSH)) and their corresponding disulfides to form a mixed disulfide at Cys-34. The resulting oxidized HMA is designated as human non-mercaptalbumin-1 (HNA-1). (2) Alternatively, Cys-34 reacts with several reactive oxygen and nitrogen species and is initially oxidized to sulfenic acid, forming one of the human non-mercaptalbumin-2 (HNA-2) isoforms. (3) Sulfenic acid reacts with thiols to form HNA-1. (4) Sulfenic acid is further oxidized by reactive oxygen species (ROS) to sulfinic and sulfonic acid. These oxidized HSA isoforms are named HNA-2 [30]. Reproduced from *Tabata* et al. [30] under the Creative Commons Attribution (CC BY) license.

**Table 1:** Overview of the mentioned post-translational modifications present on human serum albumin.

Modification/PTM	Main Site(s)	Cause/Source	Functional Consequence	Biological Relevance
Cys-34 oxidation (HNA)	Cys-34	Oxidative stress, storage	↓ Antioxidant activity; altered ligand binding; ↑ affinity for proatherogenic lipids; ↓ affinity for bilirubin, tryptophan, warfarin, and diazepam	CKD, cirrhosis, diabetes
Glycation	Lys residues; Sudlow I/II	Hyperglycemia, heat, storage	Alters charge, conformation, and drug binding; ↑ half-life; AGE formation	Diabetes, CAD, neuropathy
Carbonylation	Multiple residues	Oxidative damage	Irreversible loss of function; ↑ VCAM-1/ICAM-1; may promote atherogenesis	Oxidative stress, vascular disease
Aggregation; Polymerization	Intermolecular	Pasteurization, unfolding	Reduced ligand accessibility; altered PK; may pose immunogenic risks	Product quality concern; liver disease
Palmitoylation	Fatty acid binding regions	Lipid acylation [45]; origin unknown in product	May alter fatty acid binding	Functional modulation
Farnesylation; Geranylation	Sudlow II vicinity	Prenylation [45]; origin unknown in product	May interfere with drug/small-molecule binding	Potential transport changes
Myristoylation	C-terminus	Lipid acylation [45]; origin unknown in product	May affect protein stability	Functional modulation
Glycosylation	Multiple sites	Glycan addition [46]; origin unknown in product	↑ Half-life; altered tissue distribution/clearance	PK effects
Carbamylation	Amino groups	Uremia (CKD)	Structural alteration; amyloidogenic aggregation	CKD-associated
Guanidylation	Multiple sites	CKD	Reduced ligand-binding capacity	CKD-associated

Abbreviations: Cys-34, cysteine-34; HNA, human non-mercaptalbumin; CKD, chronic kidney disease; Lys, lysine; AGE, advanced glycation end-product; CAD, coronary artery disease; VCAM-1, vascular adhesion molecule-1; ICAM-1, intercellular adhesion molecule-1; PK, pharmacokinetics; ↓ decrease in property; ↑ increase in property.

**Table 2:** Comparison of various methods for albumin production.

Method	Purity (%)	Advantages	Disadvantages	Clinical/Industrial Application
Plasma fractionation	96	Low cost, accessible	Protein denaturation, high-quality materials needed	✓ ✓
Cohn + LC	99	Low cost, high purity and yield	Protein denaturation and/or aggregation during the addition of ethanol	✓ ✓
Human placenta blood	97.1	Minimal resin used in the process, high purity	Lesser yield, high concentration of alkaline phosphatase, restriction in blood supply	✓ ✓
Affinity precipitation	93.6	Tolerable purity, single step	Protein denaturation	× ×
Ammonium sulfate + LC	N/A	Sufficient purity, suitable for laboratory approaches	Not suitable for clinical approaches	× ✓
IEC	≤95	High yield, adsorption capacity, and recovery, convenient for large-scale production	Capital costs, ligand leakage, HSA–sorbent interactions, slow separation	✓ ✓
SMB	96	Efficient, high purity and yield	High complexity and maintenance costs	× ×
Dye ligand AC	≈98	High purity and yield, quick separation, medium specificity	Low adsorption capacity, harsh elution condition, incomplete regeneration	× ×

Abbreviations: LC: liquid chromatography, IEC: ion-exchange chromatography, SMB: simulated moving bed chromatography, N/A: not available, AC: affinity chromatography, ✓ suitable for clinical/industrial applications, × unsuitable for clinical/industrial application Adapted from *Raoufinia* et al. [20] under the Creative Commons Attribution (CC BY) license.

**Table 3:** Overview of the properties and differences of plasma-derived human serum albumin and recombinant human serum albumin.

Plasma HSA	Recombinant HSA
Derived from pooled plasma	Expressed in controlled host systems
Donor-dependent heterogeneity	Lower host- and process-controlled heterogeneity
Batch-to-batch PTM variability	Controlled host-cell- and process-dependent PTMs
Potential residual plasma components or pathogen-related material; controlled by purification and viral inactivation	No plasma-derived residuals; potential host-cell components and process-related impurities are controlled by purification
More affordable [52]	Less affordable [54]

## Data Availability

No new data were created or analyzed in this study. Data sharing is not applicable to this article.
